# Expression and regulation of the neutral amino acid transporter B^0^AT1 in rat small intestine

**DOI:** 10.1371/journal.pone.0184845

**Published:** 2017-09-15

**Authors:** Julia Jando, Simone M. R. Camargo, Brigitte Herzog, François Verrey

**Affiliations:** Institute of Physiology, Zurich Center of Integrative Human Physiology and NCCR Kidney.CH, University of Zurich, Zurich, Switzerland; Rosalind Franklin University of Medicine and Science, UNITED STATES

## Abstract

Absorption of neutral amino acids across the luminal membrane of intestinal enterocytes is mediated by the broad neutral amino acid transporter B^0^AT1 (SLC6A19). Its intestinal expression depends on co-expression of the membrane-anchored peptidase angiotensin converting enzyme 2 (ACE2) and is additionally enhanced by aminopeptidase N (CD13). We investigated in this study the expression of B^0^AT1 and its auxiliary peptidases as well as its transport function along the rat small intestine. Additionally, we tested its possible short- and long-term regulation by dietary proteins and amino acids. We showed by immunofluorescence that B^0^AT1, ACE2 and CD13 co-localize on the luminal membrane of small intestinal villi and by Western blotting that their protein expression increases in distal direction. Furthermore, we observed an elevated transport activity of the neutral amino acid L-isoleucine during the nocturnal active phase compared to the inactive one. Gastric emptying was delayed by intragastric application of an amino acid cocktail but we observed no acute dietary regulation of B^0^AT1 protein expression and L-isoleucine transport. Investigation of the chronic dietary regulation of B^0^AT1, ACE2 and CD13 by different diets revealed an increased B^0^AT1 protein expression under amino acid-supplemented diet in the proximal section but not in the distal one and for ACE2 protein expression a reverse localization of the effect. Dietary regulation for CD13 protein expression was not as distinct as for the two other proteins. Ring uptake experiments showed a tendency for increased L-isoleucine uptake under amino acid-supplemented diet and *in vivo* L-isoleucine absorption was more efficient under high protein and amino acid-supplemented diet. Additionally, plasma levels of branched-chain amino acids were elevated under high protein and amino acid diet. Taken together, our experiments did not reveal an acute amino acid-induced regulation of B^0^AT1 but revealed a chronic dietary adaptation mainly restricted to the proximal segment of the small intestine.

## Introduction

Proteins in ingested food are hydrolyzed to small oligopeptides and amino acids on their way from the oral cavity to the small intestine where they are absorbed across the mucosa. More precisely, they are transported mostly across epithelial cells by being first imported through their luminal membrane and sequentially exported across their basolateral membrane in order to be distributed to other body tissues. Also prevention of loss of amino acids via urine is necessarily important and is ensured by their reabsorption in the renal proximal tubules [1]. Intestinal absorption and renal reabsorption across membranes requires membrane-spanning transporters. These are classified according to structural similarity in groups of transporters which form solute carrier families (SLC) [2]. The SLC6 family for example first included sodium and chloride dependent neurotransmitter transporter, but later it has been shown that a cluster of formerly “orphan” transporters within this family are actually amino acid transporters including B^0^ [3]. Most neutral amino acids are transported across the apical brush-border membrane of the small intestine and the renal proximal tubules by the luminal Broad neutral Amino acid Transporter B^0^AT1 (Slc6a19), which was first identified in 2004 in mice [1, 3, 4, 5]. B^0^AT1 function has been investigated by electrophysiological experiments in *Xenopus laevis* oocytes, which revealed an electrogenic sodium co-transport for all neutral proteinogenic amino acids with the highest affinity for branched-chain amino acids and L-methionine [5, 6]. Malfunction of this transporter due to mutations in the *SLC6A19* gene causes Hartnup disorder, an autosomal recessive condition which is characterized by a urinary loss of neutral amino acids [7, 8]. In Slc6a19 nullizygous mice the sodium-dependent uptake of L-leucine was completely abolished in kidney and intestine and even the sodium-dependent glucose uptake was reduced [9].

Expression and function of B^0^AT1 have also been shown to depend on co-expression of members of the Renin Angiotensin System (RAS), namely TMEM27 (collectrin) in kidney and angiotensin converting enzyme 2 (ACE2) in small intestine [10, 11]. In ACE2 nullizygous mice, B^0^AT1 was absent in the intestinal but present in the renal brush-border membrane and in collectrin nullizygous mice B^0^AT1 was absent in the renal but present in the intestinal brush-border membrane, demonstrating a tissue-specific necessity of either of these auxiliary proteins for B^0^AT1 trafficking to the apical membrane and surface expression [11]. ACE2 is an abundant carboxypeptidase of the small intestine and has been identified as the severe acute respiratory syndrome receptor [12, 13]. It is also well known for its important angiotensin II-degrading function in the context of the renin-angiotensin system and has been shown to be involved in heart and kidney pathologies [14].

Immunoprecipitation and native electrophoresis of intestinal brush-border membrane proteins revealed that B^0^AT1 forms not only complexes with ACE2 but also with aminopeptidase N (CD13) [15]. Experimental evidence suggesting the association of peptidases and transporters in the brush-border was already reported earlier, for instance the significant reduction of sodium-dependent L-alanine transport upon removal of CD13 in bovine renal brush-border membrane vesicles (bbmv) by papain treatment [16]. CD13 is the most abundant peptidase in the mammalian small intestine [17]. It is a zinc metalloprotease that hydrolyses small oligopeptides to single amino acids at the brush-border membrane with a broad specificity for neutral amino acids [18, 19]. B^0^AT1 apparent affinity for its substrate amino acids was shown to be increased by CD13 possibly by increasing the local substrate concentration [15].

As Ferraris and Diamond postulated already in 1989, transporters should be repressed if costs exceed their benefits [20]. In general, nutrient transporters have to adapt to various dietary conditions, development and disease states [21, 22]. There is however yet not much known about the dietary regulation of amino acid transporters in contrast to that of the di- and tripeptide transporter PepT1 (SLC15A1). This proton/peptide co-transporter is transporting small oligopeptides and additionally a range of peptide-like drugs through the brush-border membrane of intestinal epithelial cells [23, 24]. Previous studies revealed different mechanism of regulation including changes on transcriptional level, mRNA stability or trafficking to the apical membrane [22].

*In vivo* studies with mice exposed to different protein diets illustrated that PepT1 function is highest in the jejunum but its strongest dietary regulation takes place in duodenum and jejunum [25]. In contrast, highest regulation of PepT1 mRNA levels was observed in the distal region of the small intestine [26, 27]. Investigation of the dietary regulation of PepT1 revealed that increased dipeptide transport activity is caused by transcriptional activation of the PepT1 gene by selective amino acids and dipeptides. Furthermore, it was shown that not only the peptide transport is increased during high protein diet but also activities of brush-border membrane enzymes and neutral aminopeptidases [28].

The few published studies about dietary regulation of amino acid transporters have revealed for instance that a high protein diet leads to an increased uptake of some amino acids and to an increased mRNA level of the apical Excitatory Amino Acid Transporter 3 (EAAT3, SLC1A1) [26, 29]. Also a glutamine-enriched diet was shown to increase glutamine uptake in jejunal bbmv from rats compared to a control diet [30]. Some experiments in which high concentrations of amino acids were administered in small intestinal loops of rats also suggested the possibility of a short-term (20 min) amino acid-induced regulation of amino acid absorption [31]. Rodents are nocturnal animals with their active phase in the night. Studies investigating a possible diurnal rhythm of PepT1 expression and function revealed variations in mRNA, protein and transport function in duodenum and jejunum, but not in ileum [32].

Based on the fact that protein and neutral amino acid availability fluctuates and that not much is yet known about the dietary regulation of intestinal amino acid transporters, we tested the hypothesis that B^0^AT1 function and expression in rat small intestine is acutely regulated by amino acid loads and/or chronically by diets. Furthermore, we tested the potential co-regulation of the associated peptidases ACE2 and CD13 along the small intestine also taking a possible diurnal regulation into account.

## Materials and methods

### Animals

All experiments were performed with male Wistar rats (Janvier, France and Charles River, Germany) that were handled in accordance with the Swiss federal and cantonal law and with the approval of the Cantonal Veterinary Office Zürich. Rats were group-housed and adapted to housing conditions for at least 1 week prior to experiments.

### Small intestine segments

The first centimeter of the small intestine after the stomach was discarded and the next 10 cm taken as duodenum. Similarly, the last centimeter before the caecum was discarded and the 10 cm before defined as ileum. The middle 10 cm of the remaining small intestine was taken as middle jejunum, whereas 10 cm in the middle of the remaining proximal and distal parts were defined as proximal and distal jejunum, respectively.

### Gastric emptying measured by *in vivo* computed tomography (CT)

Measurements of gastric emptying by *in vivo CT* were performed as previously described elsewhere [33]. Briefly, 4 h food-deprived rats received an intraperitoneal Zoletil injection (20 mg/kg, Virbac; Leuven, Belgium) and subsequently an intragastric application of 2 ml of sodium diatrizoate hydrate (200 mg/ml, Sigma-Aldrich, Steinheim, Germany) solubilized in water or in an amino acid cocktail. These 2 ml isomolar amino acid cocktail contain 0.34 mmol of each of the proteinogenic amino acids which in the case of glutamic acid corresponds to 50 mg and in terms of total amino acids to approximately 25% of the mean daily intake. Rats were imaged immediately and every 10 min for 90 min using Quantum FX 2.2 micro-CT (Perkin Elmer, Waltham, MA, USA). Image analysis was performed using Caliper Analyse 11.0 (Analyse Direct, Stilwell, KS, USA).

### Acute regulation of B^0^AT1

Rats were fed ad libitum a standard diet (3436 Kliba Nafag, Kaiseraugst, Switzerland). On the day of experiment, animals were either starved for 4 h, 16 h or fed ad libitum followed by an intragastric application of the amino acid cocktail (as described before) or water. After 1 h, rats were anesthetized with Attane^TM^ Isoflurane ad us. vet. (Piramal Healthcare, India) and euthanized by heart cut. Amino acid uptake experiments of the middle jejunum have been performed as described below. Brush-border membrane vesicles (bbmv) were prepared from 2 cm scraped mucosa of the proximal and distal jejunum for Western blot analysis as described below.

### Diurnal expression of B^0^AT1

Rats were housed under artificial 12:12-h light-dark cycle and fed ad libitum a standard diet (3436 Kliba Nafag, 18.5% protein content, Kaiseraugst, Switzerland). Three hours after light onset (Zeitgeber time 3, ZT3) and 3 h after light offset (Zeitgeber time 15, ZT15), rats were anesthetized with Attane^TM^ Isolfurane ad us. vet. (Piramal Healthcare, India) and euthanized by heart cut. Quantitative real-time PCR of RNA extracted from duodenal and ileal scraped mucosa was performed as well as Western blot analysis of proximal and distal jejunal bbmv proteins and amino acid uptake experiments with proximal and distal sections of the jejunum.

### Diets

Rats were fed ad libitum a standard normal protein diet (NP, 18% casein (AIN-93G)), a high protein diet (HP, 45% casein) or a standard diet with supplemented single amino acids (AA) in the same ratio as in the HP diet. All diets were isocaloric via adjustment of the starch content and produced by Kliba Nafag (Kaiseraugst, Switzerland). Experiments were performed after 7 days of diet treatment.

### Brush-border membrane vesicle preparation

Brush-border membrane vesicles (bbmv) were prepared following the microscale preparation protocol published by S.P. Shirazi-Beechey [34] with few modifications. Scraped mucosa from 2 cm of the small intestine was homogenized in 600 μl buffer 1 containing 100 mM mannitol and 2 mM Hepes/Tris pH 7.1 supplemented with Protease Inhibitor Cocktail (Sigma Aldrich, Steinheim, Germany). Homogenization was performed at 6 000 rpm for 20 sec with MagNa Lyser Green Beads (Roche Diagnostics, Mannheim, Germany) using the Precellys24 (BIOLABO scientific instruments, Chatel-St-Denise, Switzerland). Immediately after homogenization, 600 μl of chilled buffer 1 was added to the samples followed by magnesium precipitation (10 mM final concentration) at 4°C on a rotary mixer. After centrifugation at 3 000 g for 15 min at 4°C, supernatant was transferred to a new tube and centrifuged at 30 000 g for 30 min at 4°C. The pellet was suspended in 200 μl buffer 2 containing 300 mM mannitol, 20 mM Hepes/Tris, pH 7.4 supplemented with Protease Inhibitor Cocktail (Sigma Aldrich, Steinheim, Germany) and again centrifuged at 30 000 g for 30 min at 4°C. The final pellet was resuspended in 100 μl buffer 2 and homogenized by passing at least 10 times through a 25-gauge needle. Bbmv were either used immediately for protein quantification or snap frozen and stored at -80°C.

### Alkaline phosphatase activity

Alkaline Phosphatase activity was analyzed in bbmv and the homogenized tissue with the Alkaline Phosphatase Assay kit (Colorimetric) (abcam, Cambridge, UK) according to the manufacturer’s instructions.

### Western blotting (WB)

Protein concentration of bbmv was determined by Pierce^®^ BCA Protein Assay (Thermo Scientific, Rockford, IL, USA), following the manufactures guidelines. Bbmv (3 μg protein) were diluted in 4x Laemmli buffer supplemented with 10% β-mercaptoethanol and resolved by SDS-PAGE on 8% polyacrylamide gels. After electrophoretic transfer of proteins to a polyvinylidene difluoride (PVDF) membrane (Immobilion^®^-P, Merck Millipore, Darmstadt, Germany), nonspecific binding sites were blocked for 1 h at room temperature with 2% Top-Block^TM^ (LuBioScience, Lucerne, Switzerland) in Tris-buffered saline supplemented with 0.1% Tween-20 (TBS-T). PVDF membranes were incubated overnight at 4°C with the primary antibody diluted in 2% Top-Block^TM^-TBS-T. After washing the membranes with TBS-T, secondary antibodies diluted in 2% Top-Block^TM^-TBS-T were applied for 1 h at room temperature. Antibody binding was detected with Luminata^TM^ Classico Western HRP substrate (Merck Millipore, Darmstadt, Germany) or CDP-*Star* (Roche diagnostics, Mannheim, Germany) and visualized with FujiFilm Las-4000 camera (GE Healthcare, Glattbrugg Switzerland) according to the manufacturer’s instructions. Image J software (National Institutes of Health, Bethesda, MD, USA) has been used for densitometric analysis of Western blots.

### Organ fixation

After diet treatments, rats were anesthetized with Attane^TM^ Isolfurane ad us. vet (Piramal Healthcare, India) and euthanized by a heart cut. The small intestine was harvested, washed and flushed with phosphate buffered saline (PBS). Sections of 1 cm were incubated overnight at 4°C in 3% paraformaldehyde solution. After washing with PBS, samples were incubated in 30% sucrose for 8–12 h at 4°C and then for 6 h in a 1:1 mix of 30% sucrose and OCT Embedding Matrix (CellPath, Biosystems, Muttenz, Switzerland). Subsequently, samples were embedded in OCT Embedding Matrix and stored at -80°C.

### Immunofluorescence (IF)

Serial sections of 5 μm were cut on a cryostat, collected on Superfrost Plus slides (Gerhard Menzel B.V. & Co. KG, Braunschweig, Germany) and stored at -80°C. Sections were incubated in pure methanol for 90 sec at -20°C and then rehydrated for 15 min at room temperature. After washing in phosphate-buffered saline (PBS), blocking was performed with PBS-2% BSA-0.04% Triton X-100 for 1 h followed by incubation with the primary antibodies (diluted in PBS-2% BSA-0.04% Triton X-100) overnight at 4°C. Sections were then washed three times with PBS and subsequently incubated with the secondary antibodies (diluted in PBS-2% BSA-0.04% Triton X-100) for 1 h at room temperature. To counterstain the nuclei, 4’,6-Diamidino-2-Phenylindole Dihydrochloride (DAPI, Thermo Fischer Scientific, Waltham, MA, USA) was added to the secondary antibody mixture (1:5 000 dilution). After being washed three times with PBS, sections were mounted using DAKO Glycergel Mounting medium (Dako North America, Capinteria, CA, USA). Analysis has been performed with the automated upright confocal laser scanning microscope SP8 (Leica) and image analysis has been performed with Imaris (Bitplane) software at the Center for Microscopy and Image Analysis (ZMB) of the University of Zürich.

### Antibodies

The following primary antibodies were used: rabbit anti-mouse B^0^AT1 antibody ([3], 1:100 for IF, 1:2 000 for WB), goat anti-mouse ACE2 (R&D Systems, Minneapolis, MN, USA; dilution: 1:100 for IF, 1:1 000 for WB), rabbit anti-human CD13 (abcam, Cambridge, UK; dilution: 1:100 for IF, 1:2 000 for WB), and mouse anti-β-actin (Sigma Aldrich, Steinheim, Germany; dilution: 1:100 for IF and 1:5 000 for WB). Secondary antibodies were Anti-Rabbit IgG HRP Conjugate, Anti-Mouse IgG (H+L) AP Conjugate (Promega, Dübendorf, Switzerland) or donkey anti-goat IgG-HRP (Santa Cruz Biotechnology, Dallas, Texas, USA) for Western blot analysis (1:5 000 dilution) and Alexa Fluor^®^ 594 donkey anti-rabbit IgG (H+L) (life technologies, Carlsbad, California, USA) and Dnk pAb to Goat-IgG (DyLight^®^ 488) (abcam, Cambridge, UK) for immunofluorescence (1:2 000 dilution).

### Amino acid uptake

Compared to Na^+^-dependent glucose transport, intestinal bbmv displayed little and variable Na^+^-dependent neutral amino acid transport activity. Therefore amino acid transport studies were performed using inverted sections (rings) of small intestine. Rats were anesthetized with Attane^TM^ Isolfurane ad us. vet. (Piramal Healthcare, India) and euthanized by heart cut. The small intestine was harvested, washed and flushed with PBS and subsequently inverted. To investigate the acute regulation of B^0^AT1 ring uptake of radiolabeled L-isoleucine was performed as previously described [35]. Briefly, one to two centimeter inverted rings of the middle jejunum were incubated for 5 min at 37°C in bubbling (oxycarbon) Krebs-Tris buffer (pH 7.4) containing L-isoleucine (0.1 μCi ^14^C-L-Ile/ml) with or w/o sodium. For these experiments 1 mM L-isoleucine was used (a concentration slightly below its published K_M_ for B^0^AT1 transport [6, 9]) in order to minimize the relative contribution of the basolateral high- and low-affinity transporters to the measured uptake rate. Rings were washed on a filter system, dried o/n at 55°C and weighed. Lysis was performed for 6 h on a shaker in 1 ml Solvable^TM^ (Perkin Elmer, Waltham, MA, USA) followed by 1 h of bleaching with H_2_O_2_. Ultimate Gold^TM^ scintillation fluid (Perkin Elmer, Waltham, MA, USA) was added followed by determination of radioactivity using the liquid scintillation analyzer (Packard Tri-Carb 2900TR, PerkinElmer, Waltham, MA, USA). To investigate the chronic effect of diets on B^0^AT1 function, 1 cm ring sections of the proximal and distal jejunum were used for transport studies. Ring sections were slid onto 2 ml-serological pipettes (Sarstedt, Nümbrecht, Germany) which were placed in tubes filled with sodium-free Krebs-Tris buffer (pH 7.5) stored on ice. Uptake of 1 mM L-isoleucine (0.1 μCi ^14^C-l-Ile/ml) was performed in Krebs-Tris buffer (pH 7.5) in the presence and absence of sodium at 37°C for 2 min. The uptake was stopped by washing the ring sections in ice-cold sodium-free Krebs-Tris buffer (pH 7.5). After lysis of the tissue overnight on a shaker in 1 ml Solvable^TM^ (Perkin Elmer, Waltham, MA, USA), Ultimate Gold^TM^ scintillation fluid (Perkin Elmer, Waltham, MA, USA) was added followed by determination of radioactivity using the liquid scintillation analyzer (Packard Tri-Carb 2900TR, PerkinElmer, Waltham, MA, USA).

### Quantitative real-time PCR

Total RNA from scraped mucosa was isolated using RNeasy Mini Kit (Qiagen, Hilden, Germany), following the manufacturer’s instructions. Reverse transcription of the isolated RNA was performed with TaqMan^®^ Reverse Transcription Kit (Thermo Fischer Scientific, Waltham, MA, USA) followed by quantification of the gene expression by quantitative real-time PCR using SYBR Green JumpStart^TM^ Taq ReadyMix^TM^ (Sigma-Aldrich, Steinheim, Germany) according to manufacturer’s instructions. Primers are listed in Table 1.

**Table 1:** List of primers used for qPCR.

Target mRNA	Primer sequence
B^0^AT1	Sense: GCCACCGTGGTCTACTCTATCATT Antisense: GGCAGGTCGAACCCATTG
ACE2	Sense: GGACCGACAACGAAATGTACCTA Antisense: GCCTCCCCAAAAGGAACTG
18S	Sense: AGGATCCATTGGAGGGCAAGT Antisense: TCCAACTACGAGCTTTTTAACTGCA

### Amino acid measurements following gavage experiments

After diet treatments, rats were starved for 12 h during the day. Three hours after light offset they received an intragastric application (1 ml per 100 g) of an amino acid mixture of all proteinogenic amino acids with a final concentration 10-fold higher than the plasma concentration (gavage-amino acid mixture, Table 2). The mixture was supplemented with 0.5 μCi/ml ^3^H-mannitol and 0.05 μCi/ml ^14^C-radiolabeled L-isoleucine. After 1 h, rats were anesthetized with Attane^TM^ Isolfurane ad us. vet (Piramal Healthcare, India) and euthanized by heart cut. The blood was collected in heparin-coated tubes (Braun Medical AG, Sempach, Switzerland), plasma was purified and 20 μl were used for ultra performance liquid chromatography amino acid measurements. The small intestine was harvested and each 10 cm of the proximal, middle and distal part were washed with 1 ml PBS. The content was collected and sections were inverted followed by mucosa scraping. The intestinal content, scraped mucosa and plasma samples were lysed overnight on a shaker in 1 ml Solvable^TM^ (Perkin Elmer, Waltham, MA, USA), followed by addition of Ultimate Gold^TM^ scintillation fluid (Perkin Elmer, Waltham, MA, USA) and determination of radioactivity using the liquid scintillation analyzer (Packard Tri-Carb 2900TR, PerkinElmer, Waltham, MA, USA).

**Table 2:** Gavage-amino acid mixture.

Amino Acid	AA mix (10x plasma)
	[mmol/ml]
L-alanine	4,190
L-arginine	2,050
L-asparagine	0,590
L-aspartate	0,270
L-cystein	0,300
L-glutamate	1,470
L-glutamine	7,310
glycine	2,300
L-histidine	0,690
L-isoleucine	0,990
L-leucin	1,740
L-lysine	4,030
L-methionine	0,600
L-phenylalanine	0,720
L-proline	1,630
L-serine	1,690
L-threonine	2,730
L-tryptophane	1,230
L-tyrosine	0,820
L-valine	2,050

Plasma concentration according to [36].

### Ultra performance liquid chromatography amino acid measurements

Analysis of the amino acid concentration was performed at the Functional Genomic Center Zurich (FGCZ) using the Mass Track Amino Acid Analysis Application Solution by ACQUITY ultra performance liquid chromatography (UPLC; Waters, Milford) according to the manufacturer’s protocol. Plasma samples were diluted 1:1 with 10% sulfosalicylic acid for deproteinization prior to UPLC.

### Statistics

Analysis of experimental data was performed with GraphPad Prism 5.0 and MS Office Excel. Statistical significance between mean values of groups was tested using paired two-tailed Student's *t*-test, one-way ANOVA or two-way ANOVA and a Bonferroni post test. Differences were considered significant at *P* < 0.05. Data are expressed as mean ± SEM.

## Results

### Distribution of B^0^AT1 and its associated peptidases along the small intestine

**Fig 1:**
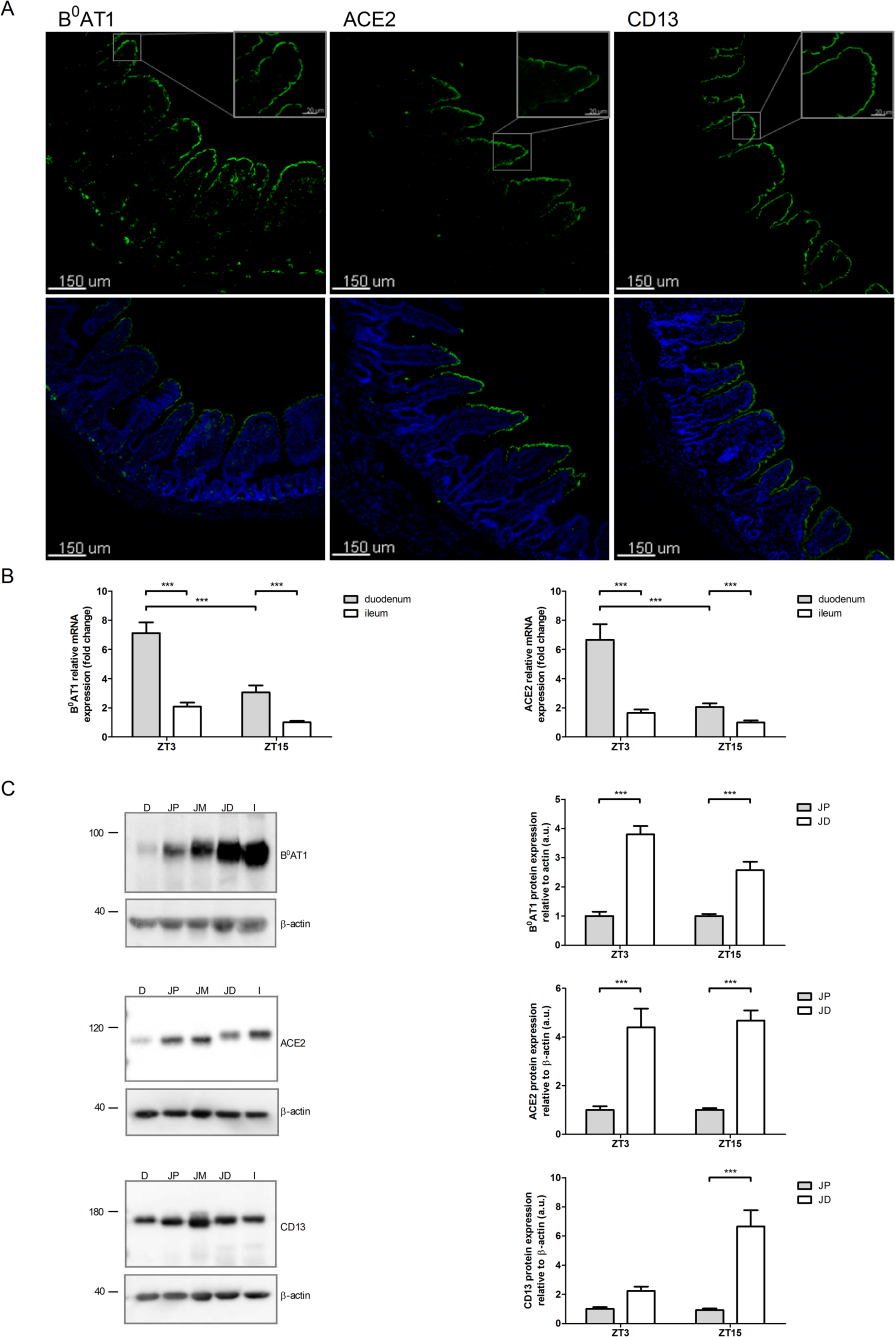
Axial expression and localization of B^0^AT1, ACE2 and CD13. A: Localization of B^0^AT1, ACE2 and CD13 by immunofluorescence analysis. Expression of B^0^AT1, ACE2 and CD13 in the ileum is visualized by confocal microscopy. Upper panel: low-and high magnification images of protein of interest; lower panel: low-magnification images of protein of interest (green) and DAPI (blue). B: Relative mRNA abundance of B^0^AT1 and ACE2 in duodenum versus ileum. The mRNA expression levels of B^0^AT1 and ACE2 were measured by quantitative real-time PCR. Rats were either euthanized 3 h after light-onset (ZT3) or 3 h after light-offset (ZT15). Scraped mucosa of duodenum and ileum was used for analysis. The level of tested mRNAs standardized to 18S rRNA and normalized to the ZT15 value in the ileum is shown. The values represent mean levels from 27 rats pooled from three independent experiments in which rats were fed either a NP, HP or AA diet. Values are expressed as means ± SEM; n = 27; two-way ANOVA and Bonferroni's post test; *** *P* < 0.001, ** *P* < 0.01. C: Protein expression of B^0^AT1, ACE2 and CD13 along the small intestine. Western blotting experiments of bbmv with antibodies directed against B^0^AT1, ACE2, CD13 and β-actin were performed. Representative Western blotting images are shown in the left panels; D = duodenum, JP = proximal jejunum, JM = middle jejunum, JD = distal jejunum, I = ileum; Molecular weight markers are indicated in kDa. The intensity of the immunoreactive bands was quantified, standardized to β-actin and normalized for each time point to JP in order to highlight the differences between JP and JD. Data shown in the right panels represent mean levels from 18–27 rats pooled from three independent experiments in which rats were fed either a NP, HP or AA diet. Values are expressed as means ± SEM; n = 18–27; two-way ANOVA and Bonferroni's post test; *** *P* < 0.001.

The localization of B^0^AT1, ACE2 and CD13 in rat small intestine was investigated by immunofluorescence. In duodenum and ileum, antibodies directed against B^0^AT1, ACE2 and CD13 showed a strong apical staining along the intestinal villi as it was shown before for mouse tissue [3, 11]. As shown for all three proteins in ileum (Fig 1A), their signal was absent in the crypts and displayed a similar gradient along the villi with an increasing intensity towards the tips.

The relative abundance of mRNAs encoding B^0^AT1 and ACE2 was measured in duodenal and ileal mucosa by quantitative real-time PCR using 18S rRNA as an internal control. Fig 1B shows the relative abundances of these transcripts in rats euthanized 3 h after light-onset (ZT3) or 3 h after light-offset (ZT15). Three independent experiments have been performed in which rats were fed either a normal protein diet (NP), a high protein diet (HP) or a normal protein diet supplemented with amino acids (AA) and data were subsequently pooled, because no dietary effect on the expression of B^0^AT1 and/or ACE2 mRNA was observed. Both transcripts showed at both times of the day a higher level in duodenum compared to ileum.

Protein abundance of B^0^AT1, ACE2 and CD13 along the small intestine was measured by Western blotting performed on brush-border membrane vesicles (bbmv) prepared from duodenum, proximal-, middle- and distal jejunum as well as ileum and quantified relative to the microvilli cytoskeletal protein β-actin,. Surprisingly, as shown in representative Western blots of Fig 1C (left panels), the abundance of these proteins displayed an axial gradient opposite to the one of the corresponding mRNAs. The enrichment of luminal proteins in bbmv used for Western blot analysis was verified by alkaline phosphatase activity measurement that indicated an 8-fold increase compared to total lysate (data not shown). Additionally, rats fed different diets (NP, HP and AA) were euthanized 3 h after light-onset (ZT3) or 3 h after light-offset (ZT15). To visualize the axial expression gradient at the two time points values measured with the different diets were pooled. The three proteins tested in these bbmv showed a clearly increasing expression level in distal direction along the small intestine, as shown for distal versus proximal jejunum at both time points (Fig 1C, right panels). Indeed, the protein expression levels of B^0^AT1, ACE2 and CD13 were at least two-fold higher in distal than proximal jejunum and their maximal increases were 5-, 9- and 13-fold, respectively.

### Circadian variation of luminal B^0^AT1 function and expression

**Fig 2:**
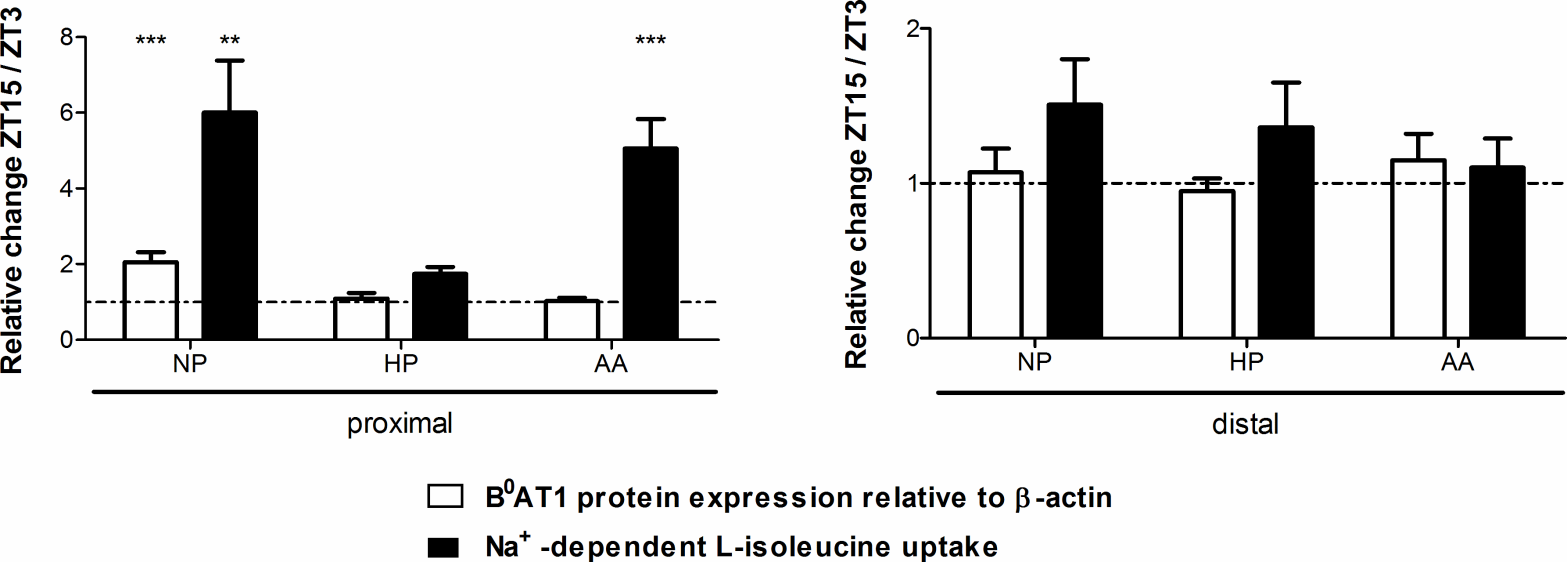
Circadian effect on B^0^AT1 protein expression and L-isoleucine uptake rate. Animals were fed NP, HP or AA diet for 7 days and euthanized either 3 h after light-onset (ZT3) or 3 h after light-offset (ZT15). Western blotting experiments with antibodies directed against B^0^AT1 and β-actin were performed with bbmv of the proximal and distal jejunum (same preparations as in Fig 1) and the intensity of the immunoreactive bands was quantified, standardized to β-actin and normalized to ZT3 in order to highlight the differences between time points. Data represent mean values of 9 different animals. Values are expressed as means ± SEM, two-way ANOVA and Bonferroni's post test; *** *P* < 0.001. The intestinal ring-uptake of L-isoleucine in proximal and distal jejunum at ZT3 and ZT15 was measured in the presence and absence of sodium and sodium-dependent uptake was calculated. Each uptake condition was measured in triplicates. Data represent mean values of 9 different animals from 3 independent experiments. Values are expressed as means ± SEM, two-way ANOVA and Bonferroni's post test, ** *P* < 0.01, *** *P* < 0.001.

Rats belong to the category of nocturnal animals with their active phase in the night and their inactive phase during the day. To investigate whether there is a diurnal change of B^0^AT1 mRNA, protein and/or function, rats fed different diets (NP, HP or AA diet) were euthanized 3 h after light-onset (ZT3) or 3 h after light-offset (ZT15). B^0^AT1 protein expression in bbmv (same preparations as in Fig 1) was measured in proximal and distal jejunum at ZT3 and ZT15 by Western blotting and signals standardized to β-actin were normalized to ZT3 values (Fig 2). As shown in Fig 1C, the distal B^0^AT1 protein expression was higher than the proximal one, independent of the diet or time point. This effect is not visible in Fig 2 due to the normalization to ZT3 in order to highlight the difference between the active and inactive phase. These experiments revealed that luminal B^0^AT1 protein expression was not significantly different between the active and inactive phase except in the proximal jejunum of rats fed a NP diet in which it was significantly increased during the active phase (ZT15) compared to the inactive one (ZT3) (Fig 2, left panel). It is not clear why this difference was observed under NP diet and not under HP or AA diet as well. Interestingly, compared to the protein expression the mRNA expression of B^0^AT1 measured in duodenum and ileum appeared to follow a time-shifted circadian regulation with a significantly higher mRNA expression at ZT3 compared to ZT15 in duodenum that was observed with all diets (Fig 1B). It might be that this timing difference represents a phase shift of the mRNA regulation that is adapted to the timing of protein translation and half-life. The transport function of B^0^AT1 was tested by measuring the uptake rate of L-isoleucine in intestinal rings. L-isoleucine belongs as a large aliphatic neutral amino acid to the preferred substrates of B^0^AT1 [5]. The results of these experiments performed with rings of proximal and distal jejunum and in the presence and absence of sodium are shown in Fig 2. In the proximal jejunum (Fig 2, left panel), the uptake rate was significantly higher at ZT15 compared to ZT3 under NP and AA diet. The same trend was shown for HP diet and also in the distal jejunum (Fig 2, right panel) independent of the diet but without significance. Both, lowest and highest transport rates were observed in proximal jejunum at ZT3 and ZT15, respectively, suggesting that the function of B^0^AT1 is more regulated in early than late segments of the small intestine.

### Delayed gastric emptying induced by intragastric application of an amino acid cocktail

**Fig 3:**
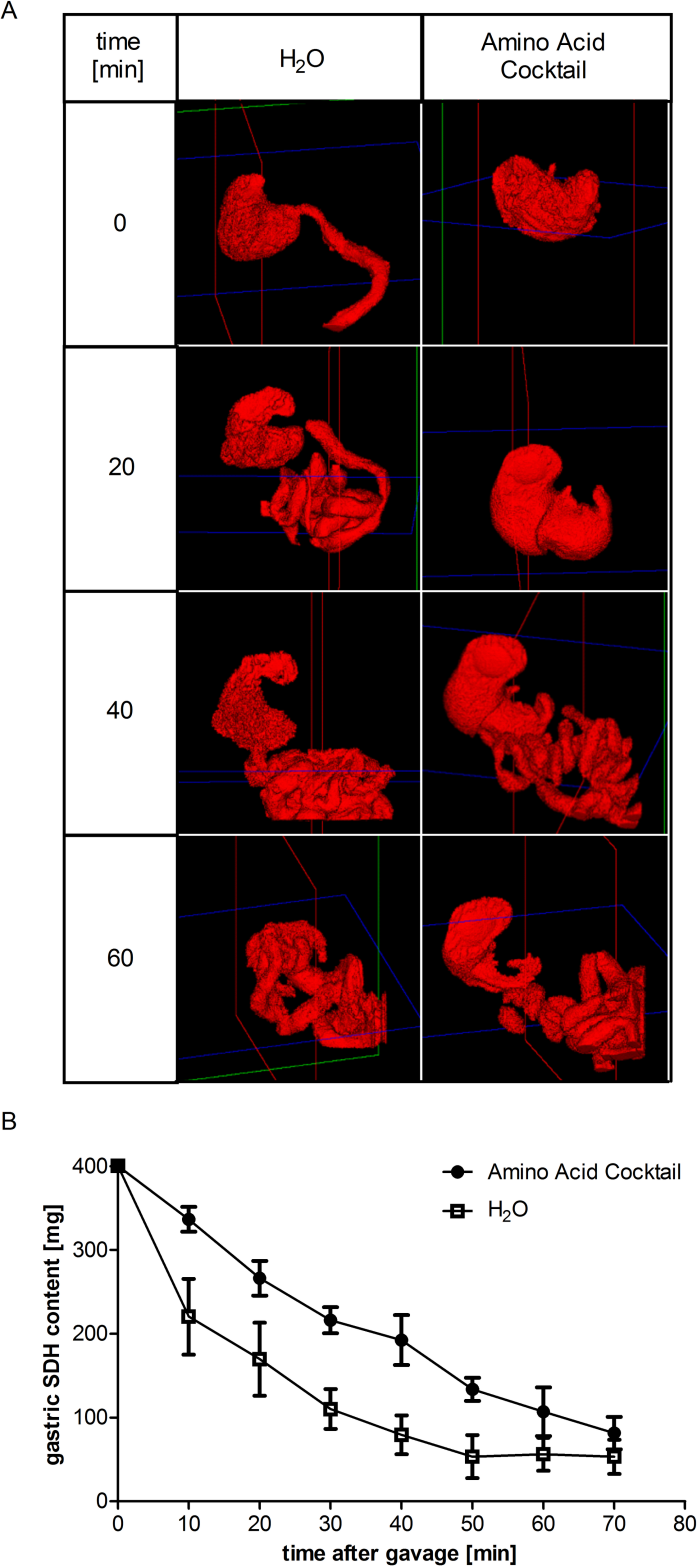
Impact of an amino acid cocktail on the time course of gastric emptying. Food-deprived rats were gavaged with contrast agent (sodium diatrizoate hydrate, SDH) and water or an isomolar amino acid cocktail and the impact on gastric emptying was measured with computed tomography (CT). Three-dimensional volume renderings of representative stomachs are shown immediately (0 min), 20, 40 and 60 min after gavage (A). Gastric SDH content was quantified over time which reflects the gastric emptying (B). Values are expressed as means ± SEM; n = 5.

To determine the time-point at which to sacrifice animals after amino acid gavage, we examined the time-course of gastric emptying and small intestinal transit upon amino acid gavage. We administered a hydrophilic contrast agent together with water or with an isomolar amino acid cocktail and measured its impact on gastric emptying by *in vivo* CT imaging in rats (Fig 3A). Quantification of the contrast agent in the stomach over time revealed a delay of gastric emptying of about 20 min with the amino acid cocktail compared to pure water (Fig 3B). The majority (three-quarter) of the contrast agent was released into the small intestine 60 min after intragastric application, a time at which no measurable amount had reached the caecum yet. For following experiments, animals were therefore euthanized at 60 min after gavage.

### Testing for acute B^0^AT1 regulation by a proteinogenic amino acid cocktail

**Fig 4:**
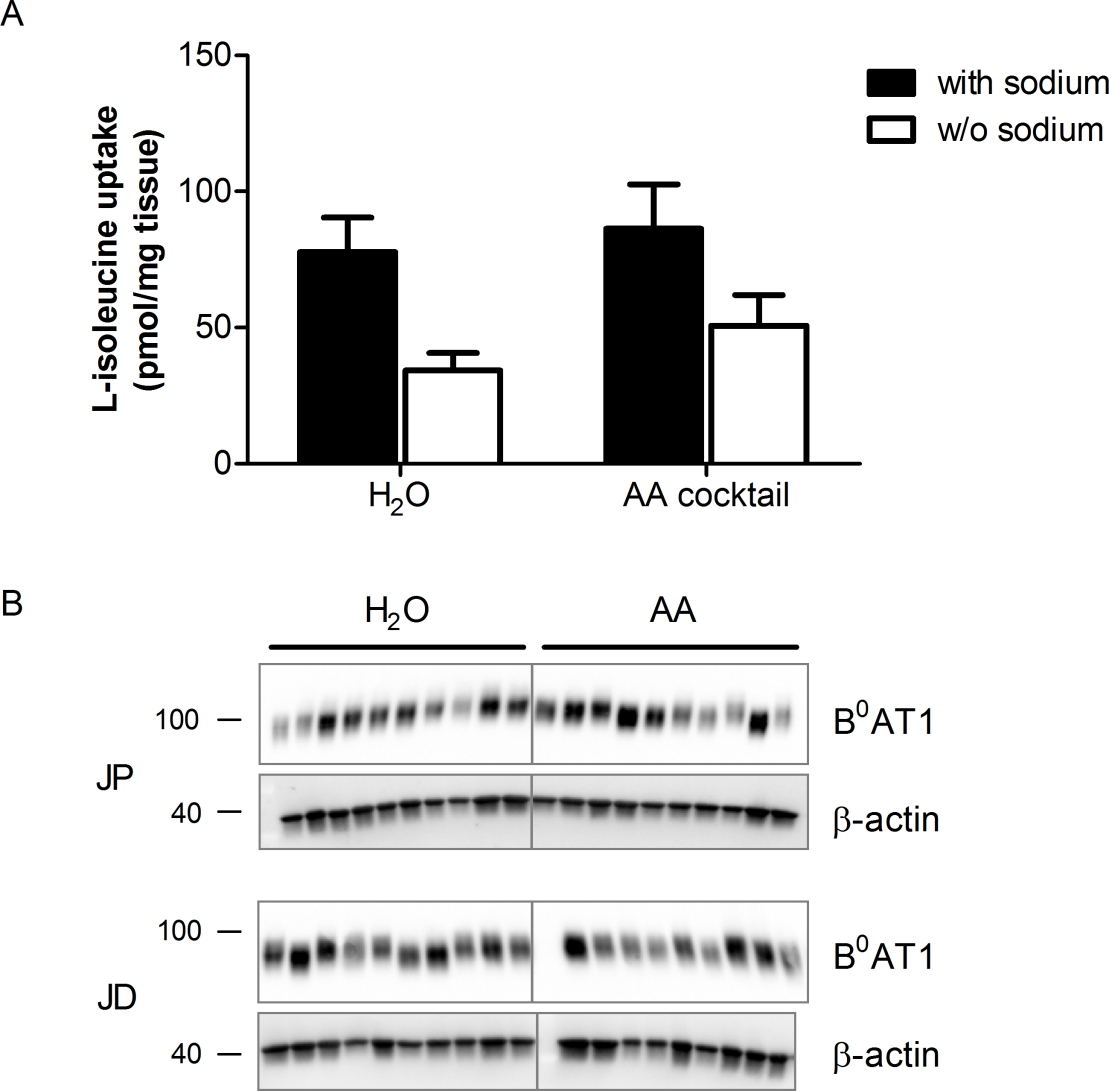
Lack of short-term effect by amino acid cocktail gavage on L-isoleucine transport and B^0^AT1 membrane expression. Food-deprived rats were gavaged with water or an isomolar amino acid cocktail. One hour after gavage, the transport of L-isoleucine into everted rings of the middle jejunum was measured in the presence (black bars) or absence of sodium (white bars) (A). Data represent mean values of 5 rats ± SEM, each uptake condition was measured in triplicates; no significant difference of sodium-dependent uptake by paired two-tailed Students t-test. B: Western blotting experiments of bbmv with antibodies directed against B^0^AT1 and β-actin were performed. Representative Western blotting images are shown. Molecular weight markers are indicated in kDa.

To investigate the possible acute regulation of B^0^AT1, rats food-deprived for 16 h were gavaged with water or an isomolar amino acid cocktail containing 0.34 mmol of each of the proteinogenic amino acids. One hour after gavage, analysis of L-isoleucine transport in ring sections of the middle jejunum was performed. Sodium-dependent uptake of radiolabeled L-isoleucine was clearly demonstrated but no difference in transport between animals receiving water or amino acid cocktail was observed (Fig 4A). Analysis of protein expression in bbmv of proximal and distal jejunum from the same rats was in line with this result displaying no obvious differences between the two groups (data not shown). Additional quantitative examinations of the B^0^AT1 protein expression in bbmv from animals starved for 4 h followed by intragastric application of water or the amino acid cocktail also showed similar values without any significant differences (Fig 4B). Furthermore, animals fed ad libitum showed similar values of B^0^AT1 protein expression. We tested these different conditions because previous studies had revealed that fasting itself can have an impact on transport rate and transporter expression. It had been shown that one day of fasting increases dipeptide transport in rat intestine by increasing the PEPT1 protein expression in the brush-border membrane and in addition 16 h of starvation of mice had been shown to cause a significant upregulation of intestinal PEPT1 protein expression [37,38].

Taken together, our results did not support the hypothesis of an acute regulation of intestinal B^0^AT1 by dietary amino acids.

### Chronic regulation of B^0^AT1, ACE2 and CD13 by different diets

To investigate the impact of different diets on B^0^AT1 regulation, rats were fed with a NP, HP or AA diet for 7 days. Three hours after light-onset (ZT3) or 3 h after light-offset (ZT15) rats were euthanized, tissues were harvested and transport measurements by intestinal ring uptakes immediately performed. The results of these experiments that aimed at revealing the impact of the different diets on mRNA and protein expression of B^0^AT1 and its auxiliary peptidases as well as on B^0^AT1-mediated amino acid transport were not straight forward but clearly demonstrated significant effects that are summarized here.

**Fig 5:**
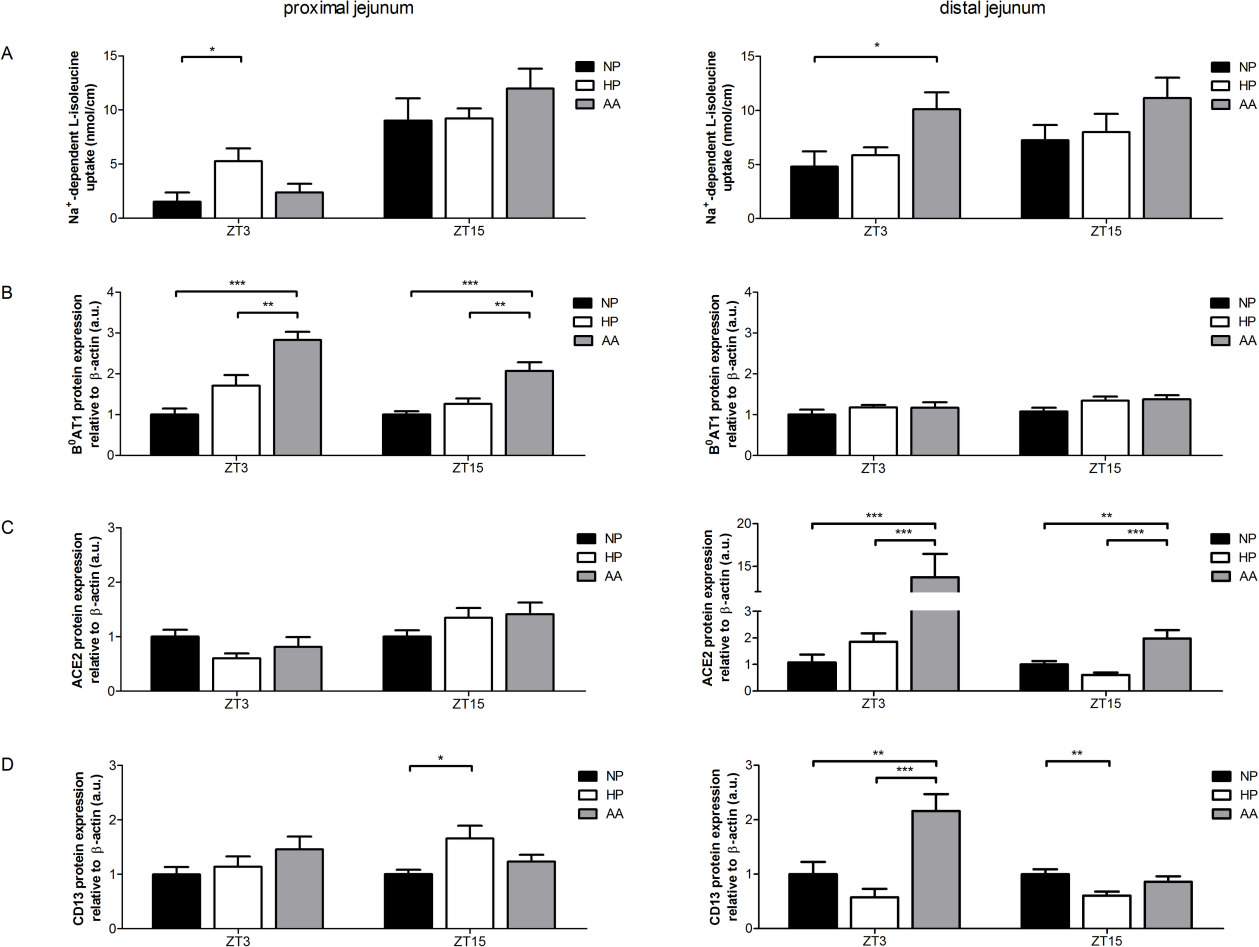
Effect of different diets on L-isoleucine transport and B^0^AT1, ACE2 and CD13 membrane expression. Animals fed NP, HP or AA diet for 7 days were euthanized 3 h after light-onset (ZT3) or offset (ZT15). A: The transport of L-isoleucine into everted rings of the proximal and distal jejunum was measured in the presence and absence of sodium and sodium-dependent uptake was calculated. Data represent mean values of 3 intestinal ring uptakes of 9 different animals tested in 3 independent experiments. B-D: Western blotting experiments with antibodies directed against B^0^AT1, ACE2, CD13 and β-actin were performed in bbmv of the proximal and distal jejunum (same preparations as in Fig 1). The intensity of the immunoreactive bands was quantified, standardized to β-actin and normalized to NP diet. Data represent mean values of 9 different animals. All values are expressed as means ± SEM, one-way ANOVA and Bonferroni’s post test was performed for each time point; * *P* < 0.05, ** *P* < 0.01, *** *P* < 0.001.

As regards the transport function, uptake was significantly higher (~3-fold) under HP diet compared to NP and AA diet in the proximal jejunum at ZT3 (Figs 2B and 5A). In contrast, at ZT15 the transport rate (that in all conditions was higher than at ZT3) was slightly increased under AA diet compared to HP or NP diet (by about 20%). In the distal jejunum, in the inactive (ZT3) as well as in the active (ZT15) phase, transport under AA diet was increased by approximately 30–40% compared to NP and HP diet (Fig 5A), indicating that also in the distal jejunum transport was adapted to the diet. In summary, the transport rate of L-isoleucine was slightly increased along the jejunum under AA and/or HP diet compared to NP diet. Since this increase was measured per unit gut length, it is not clear to what extent it may be due to tissue hypertrophy.

To evaluate whether there is also an adaptation of the expression of proteins involved in the luminal transport of neutral amino acids, protein expression of B^0^AT1, ACE2 and CD13 in bbmv (same samples as in Fig 1) was analyzed (Fig 5B–5D).

Regarding the luminal B^0^AT1 protein, its expression in the rat proximal jejunum was significantly increased under HP and AA diet in the active (ZT15) as well as in the inactive phase (ZT3) (Fig 5B). However, in the distal jejunum there was no significant change in B^0^AT1 protein expression under HP or AA diet compared to NP diet, neither at ZT3 nor at ZT15. These data suggest that there is an adaptation of B^0^AT1 protein expression to the diet in the proximal part of the jejunum but interestingly not in its distal part. In summary, luminal B^0^AT1 protein expression was increased in the proximal section of the small intestine under AA diet.

Since the two peptidases ACE2 and CD13 were shown to form functional complexes with B^0^AT1 and to alter its function [15], protein expression analysis in bbmv was performed (Fig 5C and 5D). Regarding the luminal ACE2 protein, its expression in the proximal jejunum was not significantly different between the diets, neither at ZT3 nor at ZT15 (Fig 5C). Only marginal changes with a slightly higher expression of ACE2 under HP diet and AA diet was found at ZT15. On the other hand, in the distal part of the jejunum ACE2 protein expression in bbmv was significantly increased under AA diet compared to HP or NP diet at both times correlating with the transport measurements of L-isoleucine. In summary, luminal ACE2 protein expression was increased in the distal section of the small intestine under AA diet.

Furthermore, analysis of CD13 protein expression in bbmv revealed that in the proximal jejunum at ZT3 it was only marginally increased under AA diet whereas at ZT15 it was significantly increased under HP diet compared to NP diet and to a lesser extent also compared to AA diet (Fig 5D). In the distal jejunum at ZT3, protein expression of CD13 was significantly increased under AA diet compared to NP and even more compared to HP diet. However, at ZT15 protein expression was significantly decreased under HP diet compared to NP diet and slightly decreased under AA diet compared to NP diet. In summary, luminal CD13 protein expression revealed no distinct expression pattern regarding the different diets.

Taken together, these results suggest that a diet with elevated free amino acid levels increases B^0^AT1 protein expression in the proximal jejunum and ACE2 protein expression as well as the transport by B^0^AT1 in the distal jejunum.

### Impact of the diet on *in vivo* L-isoleucine absorption along the small intestine

**Fig 6:**
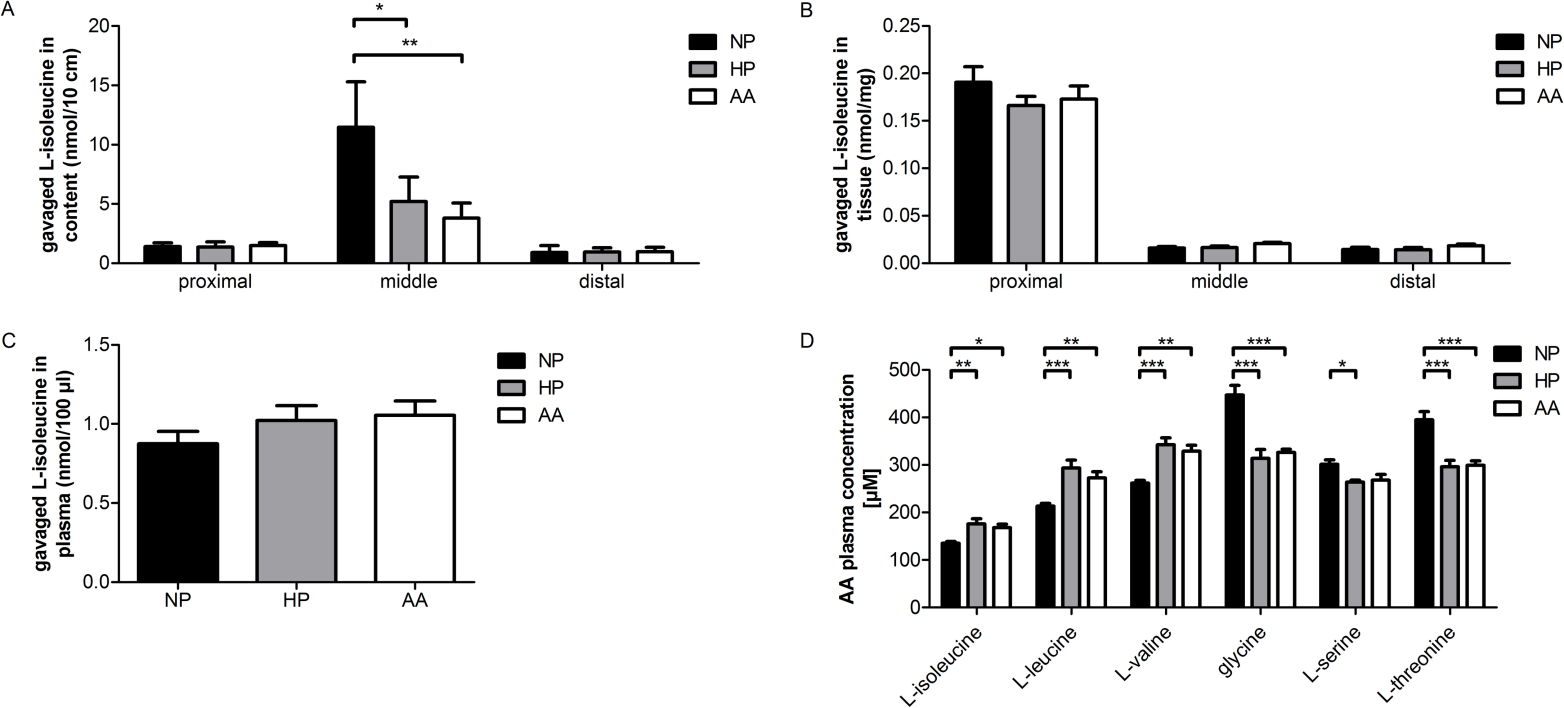
Effect of different diets on L-isoleucine absorption along the small intestine following gavage. Animals were fed NP, HP or AA diet for 7 days. An amino acid mixture (Table 2) supplemented with radiolabeled L-isoleucine was gavaged 3 h after light offset and after 1 h, the intestinal content of different segments was flushed, mucosa was scraped and blood (plasma) collected to measure radiolabeled L-isoleucine (A—C). Plasma concentrations of L-isoleucine, L-leucine, L-valine, glycine, L-serine and L-threonine were determined by UPLC. Data represent mean values of 9 different animals per group, values are expressed as means ± SEM, one-way ANOVA and Bonferroni’s post test for each amino acid; * *P* < 0.05, ** *P* < 0.01, *** *P* < 0.001.

To characterize the impact of a NP, HP or AA diet on neutral amino acid absorption along the small intestine, the luminal content in different intestinal segments, lysates of the scraped mucosa and plasma were analyzed 1 h after intragastric application of a mixture containing all proteinogenic amino acids (Table 2) supplemented with a radioactive tracer for L-isoleucine (Fig 6). The remaining amount of gavaged radiolabeled L-isoleucine in the content of the proximal and the distal part of the small intestine showed similar values under the different diets (Fig 6A). However, the remaining amount of radiolabeled L-isoleucine in the middle jejunum was significantly decreased under HP or AA diet compared to NP diet. This suggested the possibility that rats on a HP or AA diet may have expressed more functional B^0^AT1 along the small intestine and performed a more efficient absorption of neutral amino acids like L-isoleucine. However, analysis of radiolabeled L-isoleucine in cell lysates of the scraped mucosa revealed no differences between the different diets. Interestingly, the amount of radiolabeled L-isoleucine that accumulated in mucosal cells was substantially higher in the proximal part of the small intestine compared to the middle or distal part (Fig 6B). This latter observation supports the idea that free amino acids were to a large extent absorbed within the first section of the small intestine.

The amount of radiolabeled L-isoleucine found in the plasma 60 min after intragastric application did not show significantly different values between the diets but a trend of higher L-isoleucine levels under HP and AA diet compared to a NP diet (Fig 6C). This trend was supported by UPLC measurements of unlabeled amino acids in plasma shown in Fig 6D for the amino acids with significant differences between diets. Not only the concentration of L-isoleucine but also that of L-leucine and L-valine was significantly increased under HP and AA diet compared to NP diet implying that not only the newly absorbed neutral amino acids are taken up more efficiently but also the plasma concentration of branched-chain amino acids was elevated under HP and AA diet. On the other hand, the plasma concentration of glycine, L-serine and L-threonine was significantly decreased under HP and/or AA diet compared to NP diet suggesting that they were possibly consumed by increased amino acid metabolism and gluconeogenesis and/or by increased basolateral uptake into small intestinal cells in exchange for absorbed BCAAs. Taken together, these results supported the hypothesis that a diet with either elevated free amino acids or elevated protein increases the expression of functional B^0^AT1 and thereby the efficient absorption of neutral amino acids along the small intestine and also increases the branched-chain amino acid plasma levels.

## Discussion

The impact of nutrition on function and expression of the neutral amino acid transporter B^0^AT1 and its auxiliary peptidases in small intestine was investigated in this study. We could show that there was a chronic dietary effect but not an acute one. Interestingly, there are many studies about regulation of sugar or peptide transporters, but there is not much known about the dietary regulation of amino acid transporters. With this study, we shed more light on the dietary regulation for B^0^AT1.

We show here that the luminal membrane expression of B^0^AT1 and of its associated peptidases ACE2 and CD13 on villi of the small intestine of rats displays an axial gradient toward the villi tips, as previously shown in *Mus musculus* intestine [3]. Additionally, our data indicated that along the small intestine there is an axial gradient of B^0^AT1 protein expression in distal direction with highest expression in the ileum, whereas its mRNA expression displays an opposite gradient. As regards the protein expression, we specifically show that in brush-border membrane vesicles (bbmv) made from intestinal mucosa the protein level of B^0^AT1 relative to β-actin increases towards the ileum. It appears that β-actin was an adequate reference for Western blot analysis because it is a highly abundant cytoskeletal protein with a “housekeeping” structural function in brush-border microvilli. The observed axial gradient of B^0^AT1 along the small intestine might make sense considering that the proportion of single amino acids relative to peptides is higher in the distal section of the small intestine compared to the proximal one after proteins have been digested and cleaved by peptidases.

Concerning the mRNA levels of B^0^AT1 and ACE2 that showed a reverse pattern with lower expression in the ileum compared to duodenum, measurements were done using scraped mucosal cells and quantifying the mRNA levels relative to the housekeeping 18S ribosomal RNA. We had first used villin mRNA as housekeeping reference, but this turned out to be more variable between samples and segments. Also analysis of these preliminary experiments revealed that the mRNA expression of B^0^AT1 and ACE2 decreased towards the ileum when quantified relative to villin. Our observation in rat small intestine contrasts with results obtained previously in mice where B^0^AT1 mRNA expression had been quantified relative to GAPDH and had shown an increase in distal direction along the small intestine [3]. On the other hand, in humans, mRNA expression of B^0^AT1 and ACE2 relative to villin was not significantly different, rather slightly decreased in the ileum compared to duodenum [39], and in cancer patients it was slightly increased in distal direction relative to total RNA [40]. We consider it as likely that the discrepancies between mRNA and protein levels along the small intestine may be explained by axial differences in translational efficiency and protein stability. However, it is also possible that the different references used for quantification played a role.

Rats are nocturnal animals and the intestinal expression of some transporter proteins has been shown to vary diurnally. It appeared therefore likely that B^0^AT1 expression and function would be higher in the evening, when more than 70% of the food intake takes place and the animals have their active phase. Therefore we investigated transporter function and expression at two different time points, 3 h after light onset (inactive phase) and 3 h after light offset (active phase). It was for example shown in previous studies that the mRNA level of PepT1 peaks three hours before onset of the dark cycle and is followed by an increased transport function just before dark-onset [32]. On the other hand, the protein level of PepT1 showed only minimal diurnal changes. For intestinal hexose transporters it was shown that mRNA expression peaks 6–12 h earlier than protein expression [41]. Furthermore, it was shown that protein expression and uptake of glucose goes together with a peak at the beginning of the dark cycle. Our present experiments show on the one hand an increased transport rate in the evening (ZT15) and on the other hand an increased mRNA expression of B^0^AT1 and ACE2 in the morning (ZT3). The increased mRNA levels measured in the morning could, depending on the half-life of the gene products, participate to an upregulation of the corresponding protein for the evening. However, B^0^AT1 protein expression was upregulated in the evening only under normal protein diet in the proximal part of the small intestine. Nevertheless, we observed an increased L-isoleucine uptake in the active phase (ZT15) compared to the inactive phase (ZT3) independent of the diet or section of the small intestine.

Short-term dietary effects have been demonstrated in previous studies for the regulation of glucose and L-alanine transport. For instance, the jejunal glucose absorption rate was shown to be significantly increased when either glucose or maltose was present in the lower ileum [42]. In addition, it was shown that glucose transporters respond to changes in intestinal luminal glucose concentration within one hour [43]. Also the amino acid L-alanine was shown to exert an inhibitory effect on its jejunal absorption when injected in the ileum or in an adjacent distal jejunal segment. Its absorption from the ileum was shown to be similarly decreased by its presence in the jejunum [31]. Because of the description of such substrate-mediated short-term regulatory effects, we tested whether B^0^AT1 was also acutely regulated *in vivo* by its own substrates using intragastric application of an amino acid cocktail. To determine the optimal sampling time point after gavage, we analyzed gastric emptying of the amino acid cocktail compared to water and could demonstrate a delayed gastric emptying after intragastric application of the amino acid cocktail similar to the effect already shown previously in our laboratory for specific amino acids [44]. Based on these experiments, one hour delay was considered as an optimal time point for our measurements. However, we did not observe alterations in luminal B^0^AT1 protein expression or in the L-isoleucine uptake rate one hour after amino acid application. We did not observe an effect either because potentially regulatory changes were not maintained during the experimental procedure or because such a short-term regulation does not exist. Previous studies about acute dietary regulation of nutrient transporters mentioned above have been performed by *in situ* perfusion techniques using high, probably supraphysiological substrate concentrations. Thus, it might be that the lack of effect observed in our studies was due to the more physiological experimental conditions used.

As our experiments had not revealed an acute regulation of B^0^AT1 expression or transport function in the small intestine by dietary amino acids, we then tested the hypothesis that diets containing different amounts of amino acids would in a longer-term exert such a regulatory action. For glucose and oligopeptide transporters an adaptive regulation to the diet had been described previously. Specifically in rats, mice and sheep intestinal glucose transport had been shown to be increased in response to a high carbohydrate diet and also the peptide transporter PepT1 had been shown to be upregulated by dietary proteins at the transcriptional level [27, 45, 46]. Our current experiments showed that in the proximal part of the small intestine, protein expression of B^0^AT1 in the brush-border membrane was significantly increased under AA diet (Fig 5B), an effect that in *ex vivo* ring uptake experiments translated only in a non-significant increase in uptake of the B^0^AT1 substrate L-isoleucine. However, absorption experiments of L-isoleucine *in vivo* were consistent with a more efficient absorption of this B^0^AT1 substrate from the proximal and the middle part of the jejunum under HP and AA diet (Fig 6A). It is possible that the lack of significant transport increase in ring uptakes was due to a methodological limitation of this assay that might have been not precise enough to register the difference in L-isoleucine uptake. Interestingly, ring uptake experiments in contrast showed an increase of L-isoleucine uptake in the distal part of the small intestine where B^0^AT1 protein expression was not upregulated, in contrast to its accessory protein ACE2. These data suggest the possibility that ACE2 might besides its role for B^0^AT1 trafficking also impact on its function. That associated peptidases positively impact on the function of the B^0^AT1 transporter was for instance reported by Fairweather et al., who showed that the intestinal peptidase CD13 forms functional complexes with B^0^AT1 and ACE2 [15]. Since the protein expression of luminal CD13 was increased in the distal part of the small intestine under AA diet, it might also have contributed to the observed elevation of L-isoleucine absorption. Similarly, it was reported by others that intestinal brush-border membrane enzymes like neutral aminopeptidases and γ-glutamyl-transferase displayed a higher activity when rats were fed a high protein diet [28]. In the present study, we observed an increased expression of CD13 protein under HP diet at ZT15 only in the proximal section of the jejunum. The sensing and regulatory mechanisms underlying the observed dietary adaptation of small-intestinal neutral amino acid transport are not yet understood and need further investigation. Interestingly, the HP and AA diet-induced increase of *in vivo* absorption of the neutral amino acid L-isoleucine correlated with a slightly elevated postprandial L-isoleucine plasma level and a significantly increased general plasma concentration of the branched-chain amino acids L-leucine, L-isoleucine and L-valine together with a decreased concentration of glycine, L-serine and L-threonine. Indeed, the concentration of amino acids in plasma depends, next to their uptake from nutrition, on multiple other factors controlling their fluxes within the body, for instance on their metabolism and on their loss via urine, sweat and feces [47, 48]. Taken together, the results of the present study indicate that the expression and function of luminal amino acid transporter B^0^AT1 in small intestinal enterocytes is controlled by a series of superposed regulatory effects impacting on its mRNA and protein expression levels as well as on its functional state. Specifically we show here that B^0^AT1-mediated absorption of neutral amino acids is adapted not only to the diurnal rhythm but also in the long-term to the amount of dietary proteins and amino acids, particularly at the level of B^0^AT1 expression and function in proximal segments of the small intestine.
